# Motor Competence Among Irish Adolescents: An Investigation of Sex Differences and Relatedness Between Fundamental Movement Skills and Functional Movement

**DOI:** 10.1177/00315125221137182

**Published:** 2022-10-29

**Authors:** Conor Philpott, Brian Donovan, Sarahjane Belton, Diarmuid Lester, Fiona Chambers, Wesley O’Brien

**Affiliations:** 1 School of Education, Sports Studies and Physical Education, 8795 University College Cork, Cork, Ireland; 2 School of Health and Human Performance, 8818 Dublin City University, Dublin, Ireland

**Keywords:** motor competence, motor proficiency, functional movement screen™, youth, fundamental movement skills, sex

## Abstract

In prior research, Irish youth displayed poor motor competence across fundamental movement skills (FMS) and functional movements (FM). Our purpose in this study was to compare FMS and FM across male and female Irish adolescents and to determine whether there are associations between these movement domains. We collected data on 373 adolescents (178 females; *M* age = 14.38, *SD* = 0.87 years) from six Irish secondary schools, including motor competence testing of 10 FMS, and 7 FM. Overall levels of motor competence of both FMS and FM were low, and certain levels of dysfunctional movement were high. We observed significant sex-based differences in both FMS and FM, and there was a moderate association between FMS and FM that warrants further investigation. There is a need for societal intervention and policy changes to address low levels of motor competence among adolescent youth.

## Introduction

The ability to move well is theorized as integral to youth development and an essential factor in determining positive individual health behaviors during maturation (Stodden et al., 2008). Motor competence (MC) describes the ability to perform any form of goal-directed human movement (Robinson et al., 2015). Logan et al., (2018) described fundamental movement skills (FMS) as synonymous with MC (Logan et al., 2018). FMS, the “building blocks” of efficient and effective movement, are generally categorized into three domains: object-control (catching, kicking, dribbling, striking and throwing), locomotor (running, skipping, jumping for height and jumping for distance) and stability (balancing) (Logan et al., 2018; Victoria Department of Education, 1996).

The associations between FMS and physical activity (PA), improved physical fitness, and lower body mass index (BMI) indicate that FMS development contributes positively to maintaining a healthy lifestyle among children and adolescents (Barnett et al., 2022; Graham et al., 2022; Logan et al., 2015). Recent expert statements and systematic reviews have highlighted that current FMS levels among children and adolescents globally are poor, particularly in comparison to the original normative data of FMS measurement tools (Bolger et al., 2021; Duncan et al., 2022; Ulrich, 2000). FMS sex differences among childhood and adolescent global populations (with male superiority) have been frequently reported (Barnett et al., 2016; Lubans et al., 2010), with primary differences noted within the object-control subset of FMS (Bardid et al., 2016). Contemporary child research in Ireland recently documented poor levels of actual MC in FMS (Behan et al., 2019; Kelly et al., 2019), and these disconcerting levels of movement proficiency have not improved by adolescence, with average or below average levels also frequently reported in this population (Lester et al., 2017; O’Brien et al., 2016). As children have the maturational and developmental capacity to successfully perform FMS from the age of six, these results are unexpected (Gallahue et al., 2012). Furthermore, mastery of FMS is expected to occur by age 10; after this period, children commonly transition to the performance of more specialized movements that are conducive to sports, exercise, and other forms of PA that subsist across the lifespan (Gallahue et al., 2012). With FMS mastery expected of children by the age of 10, the assessment of maturational status among older childhood and adolescent groups within research is not common, though biological maturation may impact early and middle childhood groups (Charlesworth, 2016; Gallahue et al., 2012). Rectifying a trend toward low FMS ability in younger children is crucial to better societal health, as child and adolescent FMS skills and PA levels typically persist into adulthood (Engel et al., 2018).

Functional movement (FM) refers to efficient bodily motion, as characterized by adequate joint and muscle function that mitigates risk of injury and supports activities of daily living (Bergland & Laake, 2005; Cattuzzo et al., 2020; Cook et al., 2006b). FM has been deemed a component of MC, as FM ability requires mobility and the capacity to control the body when executing tasks (Duncan et al., 2017; Stodden et al., 2009). Greater FM levels have also been positively associated with health outcomes, namely lower BMI, increased quality of life, higher PA level and lower risk of falling (Bergland & Laake, 2005; Duncan & Stanley, 2012; Karuc & Mišigoj-Duraković, 2019). FM proficiency among child and adolescent youth have been previously characterized as sub-optimal (Duncan et al., 2017), with several studies in India, Europe, and North America having reported low values when compared to normative values (Abraham et al., 2015; Coker, 2018; Garcia-Pinillos et al., 2018). Notably, females have outperformed males in FM as per a recent systematic review and several specific studies (Garcia-Pinillos et al., 2018; O’Brien et al., 2022; Pfeifer et al., 2019).

Relationships between FM and FMS are rarely examined together, but both concepts relate directly to the definition of MC as “goal-directed human movement” (Robinson et al., 2015). There is also a theoretical link between FM and elements of FMS that argues for their joint inclusion within the MC domain (Ditcharles et al., 2017). FM and an ability to control one’s center of mass have been deemed essential to bipedal locomotion (Cattuzzo et al., 2020; Nesbitt et al., 2017). The mere fact that greater jumping height requires adequate knee flexion (assessed in squatting, and lunging FM assessments) and strong range of motion and flexibility of all leg joints suggests that jumping skills might be interwoven with functional capacity (Papaiakovou, 2013; Sánchez-Sixto et al., 2018). Examining the potential relationship between these concepts is important, since FM and FMS have both been associated with positive health markers such as PA and BMI, and practice of both movement constructs may be pivotal to maximizing these health benefits (Cattuzzo et al., 2016; Kaioglou et al., 2022; Lubans et al., 2010).

Prior studies examining the relationship between FMS and FM are scarce. One recent study with young adults (Silva et al., 2019), compared performances on a measure called the Functional Movement Screen^TM^ (FMS™; Cook et al., 2006a) with measures of MC (i.e., throw and kick velocity) and found a small positive correlation between overall FM and the total MC score and a moderate positive correlation in the stability domain (Silva et al., 2019). A recent paper examining 583 adolescents (56.09% female *M* age = 14.42 *SD* = .94 years) reported a small positive correlation between FM scores and performance in locomotor skills, indicating that the relationship between these variables is 7–8 times more probable than not (O’Brien et al., 2021). There remains a need for further research on the link between FM and FMS despite this early evidence and the theoretical basis for their association among adolescents. Past research examining FMS and FM proficiency among Irish adolescents demonstrated “alarmingly” low levels of both (McGrane et al., 2017; O’Brien et al., 2018).

To expand our understanding of the relationship between these constructs, we sought, in the present study, to measure current levels of FMS and FM proficiency and relatedness, differentiated by sex, amongst Irish adolescents. The hypothesis of this study was that low levels of proficiency in FMS and FM would be evident among both males and females, with females illustrating greater locomotor skills, and males displaying higher object-control skills. We expected maturity to impact on the FM score, with insignificant gender differences in FM. We also expected small to moderate associations between FMS and FM. This study extends earlier findings of low levels of fundamental and functional movement among Irish adolescents, and presents early findings of a relationship between FMS and FM.

## Method

### Overview

We gathered cross-sectional baseline data as part of a larger study that sought to evaluate the effectiveness of a physical education (PE) MC intervention in Ireland. We collected data in six Irish secondary schools over a two-week period in January and February 2019. Measurements relevant to this baseline study included measures of FMS, the FMS^TM^ and anthropometric measures of the participants’ height and weight.

Ethical Approval for this study was granted by the Social Research Ethics Committee in University College Cork (UCC Ethics log code “Log 2018–169”, November 2018). All named researchers of the school-based project were qualified secondary specialist physical education teachers, as recognized by the Teaching Council of Ireland. We obtained approval for each school’s participation in the study from the respective school principal (or deputy principal). We also obtained the physical education teachers’ consents to take part in the study. Also in accordance with the guidelines of the Declaration of Helsinki, we obtained informed parental consent and child assent from all direct participants.

### Participants and Environment

We invited 14 suburban secondary schools from Cork city in the province of Munster in Ireland to partake in the study (five socioeconomically disadvantaged mixed sex schools, five all-male schools, and four all-female schools). Invitations to schools were based on the following minimal inclusion criteria: (a) Teaching Council-Qualified physical education teachers operating within the schools; (b) schools contained first, second, and third-year class groups (age 12–16 years old); (c) students within the schools received a consecutive double period of physical education on a weekly basis totaling 80 minutes; and (d) all schools had access to a gymnasium hall. Following the school principals’ and teachers’ completion of a participation agreement, the school principal randomly selected a class from years 1–3 in each school. Schools were pair-matched prior to data collection on the following criteria: socioeconomic status (disadvantaged; non-disadvantaged); sex composition (single-sex boys; single-sex girls, mixed sex); facility characteristics (physical education hall and outdoor pitches) and school size (small: 0–299 students; medium: 300–599 students; and large: 600+ students) (Belton et al., 2019). This process resulted in three pairs, equally matched: two large single-sex boys’ non-disadvantaged schools, two single-sex medium girls’ non-disadvantaged schools, and two small mixed-sex disadvantaged schools. While the quality of the schools’ physical education programs was not assessed, the matching criteria and the schools’ standard provisions of qualified teachers, equal physical education time and a government mandated physical education curriculum indicated that the learning experiences of participating students were comparable (Philpott et al., 2020). Of 486 potential participants approached, 373 individuals (47.7% female; *M* age = 14.38, SD = 0.87 years) provided required consent/assent and participated in testing for at least one skill (uptake rate = 76.74%), with 324 full participants (45.9% female; *M* age = 14.33, *SD* = 0.85 years; age range: 12.23–16.37) available for MC data.

### Measurement Tools

**Figure 1.**
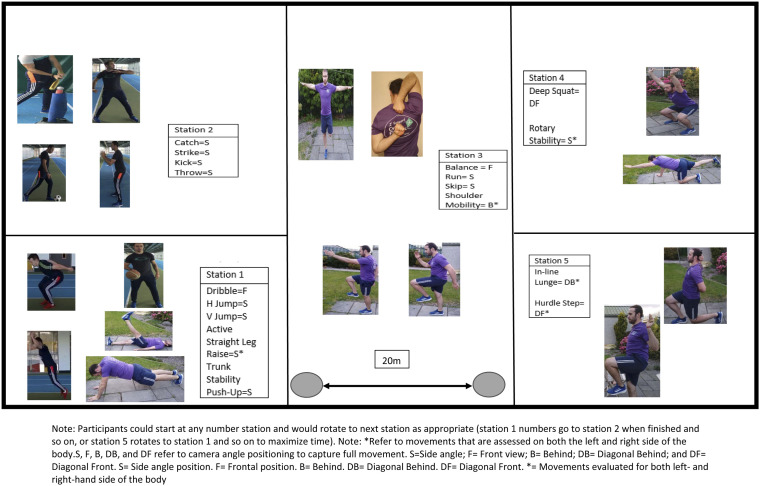
Our FMS and FMS^TM^ Station-Based Data Collection Approach. Note: Participants could start at any number station and would rotate to the next station as appropriate (station 1 numbers go to station 2 when finished and so on; or station 5 rotates to station 1 and so on to maximize time). S = Side position, F = Frontal position, B = Behind, DB = Diagonal Behind, DF = Diagonal Front, * = Movements evaluated for both left- and right-hand side of the body.

Movement measurements (FMS and FMS^TM^) were administered together during the same 120-minute physical education class, using a station-based approach. All seventeen movements (10 FMS and 7 FM) were divided among five stations that were carefully allocated to ensure equal time duration at each station, which is outlined in Figure 1. Station 1 consisted of the dribble, horizontal jump, vertical jump, active straight-leg raise, and trunk stability push-up movements. Station 2 consisted of the catch, strike, kick and throw movements. Station 3 consisted of the run, skip, shoulder mobility, and balance movements. Station 4 consisted of the deep squat and rotary stability. Station 5 consisted of the in-line lunge, and the hurdle step. Participants completed the exercises in a different order (i.e., some participants began performing movements at station 5, and when finished would go to station 1, followed by station 2 etc.) to maximize the time period allocated to researchers in schools. The station in which the participant began their assessment was primarily decided by their student code number, which was generated through class lists and the school roll system. A diagram of how the research teams prepared the stations is disclosed in the supplementary files. Participants were grouped and rotated together to each of the stations until all groups had completed each of the five stations. Utilizing recordings of these assessments, the principal investigators later scored the seventeen movement measures.

### FMS Measures

Ten FMS were tested and scored across the FMS subsets of locomotor, object-control and stability. The ten skills outlined below were selected due to their relevance in the Irish sporting and physical education context, in line with previously reported objective measurements of Irish adolescents (Issartel et al., 2016; O’Brien et al., 2016). The scoring and subsets of FMS were broken down as follows: (a) The locomotor subset (maximum score = 34) consisted of vertical jump, horizontal jump, run, and skip; (b) the object control subset (maximum score = 40) contained the catch, kick, two-handed strike, overhand throw, and stationary dribble; and (c) stability (maximum score = 10) consisted of a static balance exercise. The total maximum FMS score achievable from scores across these three subsets was 84. FMS measurements relied upon the Test of Gross Motor Development (TGMD; Ulrich, 1985) for skipping, the TGMD-2 (Ulrich, 2000) for horizontal jump, run, catch, kick, two-handed strike, stationery dribble, and overhand throw, and the Get Skilled Get Active resource for the balance and vertical jump (New South Wales Department of Education and Training, 2000; Victoria Department of Education, 1996). Construct validity and reliability of the selected FMS assessments have been established cross culturally in large samples of children and adolescents and have been reported in systemic analyses of motor assessment (Barnett et al., 2014; Issartel et al., 2016; Lander et al., 2017; New South Wales Department of Education and Training, 2000; Rey et al., 2020; Ulrich, 1985; Victoria Department of Education, 1996).

### FM Measure

To measure FM, we implemented the Functional Movement Screen™ (FMS™) (Cook et al., 2006a; 2006b). The FMS^TM^ is a physical activity pre-participation screening tool that assesses quality and function of movement to determine if individuals lack certain movement capabilities (Cook et al., 2006a; 2006b; 2014). Inter-rater reliability for the FMS™ has been previously established (Minick et al., 2010; Teyhen et al., 2012). Seven movements were assessed as part of the FMS™: active straight-leg raise, deep squat, in-line lunge, hurdle step, rotary stability, shoulder mobility and trunk stability push-up (Cook et al., 2006a; 2006b). Testing procedures were in line with the established guidelines for administering the FMS™, including the use of pre-determined verbal instructions to participants (Cook et al., 2006a; 2006b).

On the FMS™, participants received scores from 0–3 on their performances of the seven aforementioned movements (Cook, 2010). An individual’s full score for any movement was denoted as their raw score. A raw score of zero was given if the participant reported pain at any time during testing. A raw score of one was given to a participant if they were unable to complete a movement, and this was classified as a “dysfunctional” performance of the movement (Cook et al., 2014). A raw score of two was given to a participant if they were able to complete the movement using some compensations (e.g. lifting one’s heels during the deep squat). A raw score of three was given upon successful completion of the movement without any use of compensatory movements. Five of the seven movement patterns were completed bilaterally: active straight-leg raise, in-line lunge, hurdle step, rotary stability and shoulder mobility. Scores were given for each side and, if the scores were not equal, the lower of the two scores was selected to make up the participant’s ‘raw’ score for the skill. A composite FMS™ score (out of a possible 21) was derived by summing the seven raw scores together in accordance with the guidelines of screening (Cook et al., 2006a; 2006b).

### Data Collection

All field researchers involved in data collection were required to undertake two specialized training workshops totalling approximately four hours to equip them with the knowledge and skills required to accurately implement the FMS and FMS^TM^ measurement protocols. Research assistants physically practiced their assigned FMS and FMS^TM^ measurements within the training workshops to insure they could perform a demonstration, and these assistants worked with colleagues in following appropriate administration procedures, properly utilizing the verbal instructions and guidelines of the respective FMS and FMS^TM^ testing measures as applied in a previous Irish study (O’Brien et al., 2018). This research assistant training involved an objective, criteria-informed process to ensure that field staff would apply consistency to their administration and implementation of the respective FMS and FMS^TM^ measurements. Field researchers were provided with an instructional handbook at the beginning of the workshops, which outlined their roles in the data collection process and included instructions on how to implement the guidelines and measurement protocols accurately.

Participants were informed about the testing procedures for FMS (i.e. first performance being a practice performance and second and third performances being trial performances) prior to station allocation. Before each FMS performance, a field researcher demonstrated the correct technique on one occasion for the participants to observe. Feedback was not given during or after performances of the skill. These protocols are consistent with previous research in FMS (McGrane et al., 2017; O’Brien et al., 2018; Philpott et al., 2020). All movement performances were video recorded using Apple iPads (4th and 5th Gen. Apple iPad, Apple Inc, California, United States of America). We omitted data from participants with missing data due to errors regarding camera function/angle (i.e., the footage shot did not fully capture the intended movement, or the incorrect frame rate was used and therefore the performance could not be accurately assessed). These data were removed from the dataset for the individual skill wherever errors were observed, and from any composite or overall score ratings that would be generated from the associated skill.

### Data Analysis

Prior to data scoring, we established inter-rater reliability on the current sample between two principal investigators (with prior experience of data scoring) on 10% of the dataset as had been done in a prior research protocol (Lester et al., 2017; Logan et al., 2017). Inter-rater and intra-rater reliability were established using the percentage agreement method commonly utilized in Irish FMS and international research (Lester et al., 2017; McGrane et al., 2018; Ré et al., 2018). The percentage agreement was calculated by the number of rating agreements divided by the total number of rating agreements and disagreements. Two experienced raters (i.e., with two years of experience coding over 200 children across the same 17 skills as a component of another research project) double-coded 10% of the same data (*n* = 38), and the two principal investigators each scored an equal amount of the remaining dataset following the demonstration of adequate inter and intra-rater reliability.

Both the FMS and FMS™ datasets were analyzed using the Statistical Package for the Social Sciences (SPSS, v. 25.0 for Windows, IBM Corp., New York). Descriptive statistics, such as means and frequencies, for FMS and FM were prepared. Chi-square tests for independence were utilized to determine sex differences in dysfunctional movement performances. For the purpose of comparison across skills with differing maximal values (i.e. max score of 12 in vertical jump compared to max score of 10 in horizontal jump), mean raw scores were converted into percentage scores in both the FMS and FMS™ analyses. That is, the score of an individual participant in a skill was a percentage of the overall maximum score that could be obtained on the skill (e.g. a percentage value of 0.83 would be attributed to a participant who scored 10 out of a possible 12 on the vertical jump test). We used independent samples *t*-tests to determine sex-based differences in mean FMS performances. We used a one-way analysis of covariance (ANCOVA) to explore the effect of maturation on FM skill performance, with maturity offset calculated using the Moore et al. (2015) formula (Lloyd et al., 2015; Mirwald et al., 2002). Sex was an independent variable, maturity offset (i.e. age prior to or after peak height velocity) was the covariate, and the dependent variable across the ANCOVA tests consisted of scores in each FMS^TM^ movement. The ANCOVA provided results for sex-based mean differences in FMS^TM^ performances. Maturity offset and its influence on FMS performance was not examined due to the expectation that FMS are mastered by the age of 10 (Gallahue et al., 2019; O’Brien et al., 2016). Bivariate correlations between FMS and FM were calculated to determine any associations (i.e. the relatedness) between variables, with *r* = 0.10–0.29 denoting a low correlation, *r* = 0.30–0.49 denoting a moderate correlation, and *r* ≥ 0.50 denoting a strong correlation (Cohen, 1988). Statistical significance was set at *p* < 0.05, with the exception of the independent samples *t*-tests and the ANCOVA, where we applied a Bonferroni adjustment of *p *= .002 to account for multiple comparisons (8 total comparisons during the ANCOVA measurements, and 13 comparisons for the independent samples *t*-tests for a total of 21 adjustments) during data analysis. We calculated effect size using eta squared formula with eta squared values of 0.01–0.05 denoting a small effect, eta squared of 0.06–0.13 denoting a moderate effect, and eta squared ≥ 0.14 denoting a large effect size (Cohen, 1988).

## Results

Across all 17 FMS and FMS^TM^ assessments (and subsets of FMS), we obtained both inter-rater and intra-rater observer agreements of at least 95% using the percent agreement method. Descriptive data from the schools that participated are provided in Table 1 above.

**Table 1.** Participant Characteristics by School.

School	Sex	N (324)		Age		Age at PHV		FMS Raw Score (Max 84)		*FMS*^*TM*^ *Raw Score (Max 21)*	
		Male	Female	Mean	*SD*	Mean	*SD*	Mean	*SD*	Mean	*SD*
School 1	Male	63	—	14.29	0.87	13.61	0.34	66.70	5.37	12.68	1.75
School 2	Male	68	—	14.73	0.65	13.67	0.46	65.66	5.75	12.35	1.52
School 3	Female		65	14.28	0.86	12.26	0.48	63.69	6.07	12.23	1.66
School 4	Female		64	14.31	0.89	12.19	0.38	64.58	6.69	11.53	2.14
School 5	Mixed	14	5	14.14	1.02	—^a^	—^a^	62.16	7.77	11.63	2.24
School 6	Mixed	30	15	14.40	0.95	13.25	0.76	64.58	6.11	9.93	2.20

^a^Note: - Participants in this school did not consent to partaking in height and weight measurements resulting in no PHV data being compiled for this group.

### FMS Assessment Results

No participant achieved complete mastery of all FMS. The highest gross motor FMS score of any adolescent was 78 out of a score of 84, and the lowest score was 43 of 84. The overall mean composite FMS score was 64.90 (*SD* = 6.20). Having accounted for Bonferroni adjustments, an independent samples *t*-tests analysis showed no significant sex-based difference in the overall gross motor score. However, statistically significant sex differences were evident at the FMS domain level for both the object-control and locomotor skills subsets, with males scoring higher within the object-control subset; [*t*(292.01) = −7.22, *p* < .001]. However, the magnitude of the differences in the means was only moderate (eta squared = 0.13). Females scored significantly higher on the locomotor skills subset than males; [*t*(349) = 3.31, *p* < .001], however the magnitude of the differences in the means was small (eta squared = .03). Table 2 highlights FMS proficiency differences between sexes. On statistical tests, percentage values were used for analysis. At the level of individual FMS skills, males (*M *= 0.77, *SD *= 0.20) significantly outperformed females (*M *= 0.53, *SD *= 0.26) on the overhand throw [*t*(330.70) = −9.88, *p *< .001]. Males (*M *= 0.77, *SD *= 0.15) also significantly outperformed females (*M *= 0.70, *SD *= 0.17) on the kick [*t*(360) = −4.54. *p* < .001], with the effect size of differences between the means found to be large (throw; eta squared = 0.21), and small (kick; eta squared = 0.05), respectively.

**Table 2.** Mean Values (and SD) for Fundamental Movement Skill and Functional Movement Screen Proficiencies by Sex.

Item Classification	Variable	Males	Females	*p*-Values	Effect size
FMS (locomotor)	Run	0.92 (.13)	0.89 (.15)	.066	0.01
	Skip	0.73 (.24)	0.78 (.22)	.019	0.01
	Horizontal jump	0.59 (.20)	0.64 (.23)	.018	0.01
	Vertical jump	0.83 (.17)	0.88 (.15)	.004	0.02
FMS (object-control)	Catch	0.80 (.18)	0.78 (.15)	.211	0.00
	*Throw	0.77 (.20)	0.53 (.26)	.000*	0.21
	*Kick	0.77 (.15)	0.70 (.17)	.000*	0.05
	Strike	0.80 (.15)	0.78 (.18)	.258	0.00
	Dribble	0.74 (.17)	0.73 (.16)	.333	0.00
FMS (stability)	Balance	0.82 (.18)	0.81 (.16)	.503	0.00
FMS overall subsets	*Object-control	0.77 (.80)	0.70 (.10)	.000*	0.13
	*Locomotor	0.78 (.10)	0.81 (.11)	.001*	0.03
	Overall gross motor FMS	0.78 (.07)	0.76 (.08)	.019	0.02
FMS^TM^	Active straight-leg raise*	0.48 (.17)	0.65 (.21)	.000*	0.08
	Deep squat	0.48 (.20)	0.52 (.21)	.116	0.01
	Hurdle step*	0.64 (.14)	0.51 (.18)	.000*	0.15
	In-line lunge*	0.60 (.13)	0.55 (.17)	.000*	0.07
	Rotary stability**	0.58 (.14)	0.61 (.12)	.859	0.00
	Shoulder mobility	0.66 (.24)	0.67 (.24)	.826	0.00
	Trunk-stability push-up*	0.50 (.21)	0.44 (.18)	.000*	0.08
	FMS^TM^ composite score	0.58 (.09)	0.56 (.10)	.015	0.00

Note: *Denotes Significance at the Bonferroni adjusted level of *p* < .002.

Eta-squared value of 0.01–.05 denotes a small effect size. Eta-squared value of 0.06–0.13 denotes a medium effect sizes, and effect size ≥ 0.14 denotes a large effect size.

### FM Assessment Results

On the FMS™, the highest total score achieved was 17 of a possible 21, and the lowest score was 7 of 21. The overall mean descriptive (non-normalized) composite score was 12.01 (*SD* = 1.97). Percentage FMS^TM^ scores were used for the ANCOVA, correlational analyses. One-way ANCOVA tests across all seven FM on the FMS^TM^ were conducted to compare sex differences in the performance of the FMS^TM^ while controlling for the effect of maturity on participants’ performance in FMS^TM^. Maturity was not significantly related to performance on any of the seven movements (and composite score) with the exception of rotary stability [F(1, 311) = 5.323 *p <* .05]. Significant sex differences were reported on four FMS^TM^ movement performances, after eliminating the effect of maturity (Table 2). Females outperformed males on active straight-leg raise [F(1,317) = 28.103, *p* < .001 (eta squared = .08)]. Males outperformed females on the hurdle step [F(1, 318) = 56.229, *p *= .000 (eta squared = .15)], in-line lunge [F(1, 315) = 24.107, *p* < .001 (eta squared = .07)], and trunk stability push-up [F(1, 317) = 26.208, *p *= .000 (eta squared = .08)].

Chi-square testing showed that males demonstrated significantly more instances of dysfunction (raw skill scores of 1) on the active straight-leg raise (*p* < .001; phi = .35), and deep squat (*p *= .004; phi = .11) movements, whereas females had significantly more instances of dysfunction on the hurdle step, in-line lunge, and the trunk stability push-up (all at *p* < .001; phi = −.35).

### FMS and FM Associations

Bivariate correlations between FMS (overall, object-control, locomotor, and stability) and the seven FM and overall FM composite scores measured by the FMS^TM^ are depicted in Table 3. There were moderate positive correlations between the overall composite FMS and FMS^TM^ mean scores (*r* = .38, *p* < .001). A moderate correlation was also found between the overall FMS locomotor mean score and the overall FMS^TM^ composite mean score (*r* = .30, *p* < .001).

**Table 3.** Correlations (R-values) Between FMS and FM Scores.

FMS^TM^	Active Straight-Leg Raise	Deep Squat	In-Line Lunge	Hurdle Step	Rotary Stability	Shoulder Mobility	Trunk Stability Push-Up	Overall FMS™
Fundamental movement skills subsets								
Overall locomotor	.22**	.18**	.01	.14*	.12*	.17**	.15**	.30**
Overall object-control	−.07	−.03	.22**	.24**	.11	.077	.22**	.22**
Overall stability	.07	.10	.04	.15**	.19**	.14**	.10	.22**
Overall gross motor score	.10	.13*	.14*	.27**	.18**	.20**	.24**	.38**

Note: *Denotes significance at *p* < 0.05. **Denotes significance at *p* < .01; FM scores were from the Functional Movement Screen (FMS™).

## Discussion

Our goals for this study were to: (a) examine current levels of movement proficiency in terms of both FMS and FM among early adolescent Irish youth; (b) compare any sex-based differences; and (c) identify any associations between FMS and FM movement constructs that might warrant further investigation. Overall, we found low levels of FMS and FM proficiency in this large sample. High levels of FM dysfunction among adolescents were evident, with many participants having failed to successfully master the FMS that would be expected for their age cohort, and FM proficiency was below recently published normative values (Bolger et al., 2021; O’Brien et al., 2022). Most correlations between measures of FMS and FM were small, but there was a moderate correlation between overall FMS locomotor skill and the composite FM score from the FMS^TM^.

Our results indicated that these Irish adolescents showed generally poor FMS proficiency, with an overall mean FMS score of 64.90 (*SD* = 6.20) out of a possible maximum score of 84, lower than two previous samples of Irish adolescents (Lester et al., 2017; O’Brien et al., 2018). These findings are consistent with previous FMS studies of Irish adolescents that reported low levels of FMS proficiency (Duncan et al., 2022; Lester et al., 2017). Recent research has suggested a possible plateau in FMS abilities among young Irish children prior to their entry into adolescence (Behan et al., 2019; Kelly et al., 2021). While not assessed within the parameters of this study, low participation in PA by Irish children and adolescents (e.g., 17% of Irish children in primary school partaking in 60 minutes of PA a day, and 10% of secondary school children meeting this goal) has been long-documented (Woods et al., 2018). As has previously been noted in systematic research reviews, low levels of PA among children and adolescents contribute to lower levels of FMS (Graham et al., 2022; Holfelder & Schott, 2014; Logan et al., 2015).

In this study, we also found significant sex differences (see Table 2) favoring males for object-control and females for locomotor skills. We found significant sex differences on individual FMS, with males displaying superior competencies on the throw and kick, with a large effect size of 21% observed for the throw (Table 2). The throw was the only individual skill with such a significant difference and large effect size, with no other skill in the FMS section reflecting an effect size greater than small (i.e., no effect size greater than 0.05). Observed low levels of FMS proficiency amongst female adolescents in the object-control domain were also supported by previous Irish and international research (Bolger et al., 2021; Foulkes et al., 2022; Lopes et al., 2021).

Female students may not be receiving the correct pedagogy and learning experiences that would foster their development of object-control skills (Cowley et al., 2021). Trends toward higher levels of locomotor skills among girls have been commonly reported across prior Irish studies, and to a lesser extent, in international research (Behan et al., 2019; Bolger et al., 2021; Tietjens et al., 2020). Female superiority in overall locomotor skills was observed in this study (Table 2). Both female locomotor skill superiority and lower female proficiency in object-control skills have previously been attributed to sociological factors, such as influences from family, teachers, and friends (Bolger et al., 2018; Hardy et al., 2010). These sociological influences on girls and their PA patterns often culminate in females partaking in locomotor-based PA activities such as dance and gymnastics that may be viewed as more feminine (Bardid et al., 2016; Bolger et al., 2018; Humberstone, 2001).

Regarding FM, we found lower composite mean scores on the FMS^TM^ than were previously reported for children and adolescents (Abraham et al., 2015; Ghasempoor et al., 2018; O’Brien et al., 2022). We found significant sex differences on four of seven individual FM skills. Females significantly outperformed males on the active straight-leg raise, consistent with previous research (O’Brien et al., 2018; Silva et al., 2019); and, males significantly outperformed females on the in-line lunge, hurdle step, and trunk stability movements. Male superiority in the in-line lunge and trunk stability measurements has been recorded in previous adolescent and child samples (Abraham et al., 2015; Anderson et al., 2015; Garcia-Pinillos et al., 2018). Greater levels of male strength and core strength are likely to have contributed to their better performance in the skills of in-line lunge, hurdle step, and trunk stability, with the greater level of flexibility among females proving critical to their superior performance on the active straight-leg exercise (Catley & Tomkinson, 2013; Malina et al., 2015; Tomkinson et al., 2018).

Alarmingly, 91.7% of our study participants displayed a movement dysfunction (an inability to complete a movement), as denoted by a score of “1” for at least one of the seven movements assessed by the FMS™. Considering the level of dysfunctional movement observed in this Irish adolescent sample*,* it seems that our study participants may be inadequately prepared to take part in PA and/or were at risk of injury (Cook et al., 2014). Poorer FM levels have been associated with injury in prior studies; however no data from school-going children or its association with injury risk was contained within these past analyses (Attwood et al., 2019; Bonazza et al., 2017; Kiesel et al., 2007).

**Figure 2.**
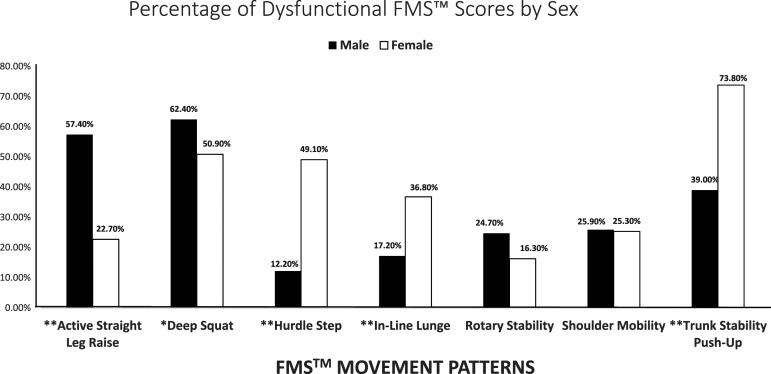
Percentage of Participants who scored a ‘Dysfunctional’ Score of ‘1,’ by Sex. Note: *Denotes Significance at the 0.05 level. **Denotes significance at the .01 level. FMS™ = functional movement screen.

Significant FM dysfunction differences between males and females (Figure 2) were observed on five of the seven FM assessments, with males significantly more dysfunctional on the active straight-leg raise and deep squat movements and females significantly more dysfunctional on the hurdle step, in-line lunge and trunk stability push-up movements. Given natural biological advantages for females in flexibility and for males in strength, sex differences appear to have impacted these performances with better female performances in the active straight-leg raise attributable to increased flexibility of females compared to males (Catley & Tomkinson, 2013; Renstrom et al., 2008; Tomkinson et al., 2018).

Notably, the associated age profiles in this study (age range 12–16) may have relevance to the earlier commencement time of puberty for females (Malina & Kozieł, 2014a, 2014b). All females in this study surpassed their peak height velocity (PHV) age, and, as calculated using the maturity offset formula, 89.7% of participants were over a year past their PHV age (Moore et al., 2015). Females often experience greater accrued gains in mobility, flexibility and neuromuscular control in the years after PHV, peaking at approximately one-year after their PHV (Ford et al., 2011; Ghasempoor et al., 2018). This may be a factor in their lower level of dysfunction in movements predicated on mobility and flexibility (Ford et al., 2011; Ghasempoor et al., 2018).

Males retained core stability and upper-body muscular strength advantages on tasks of trunk-stability push-up, in-line lunge, and hurdle step which is supported by prior findings from FM research (Abraham et al., 2015; Karuc et al., 2020). As 64% of male participants in this study had not yet reached their PHV, or were less than a year past PHV, it seems possible that some males may have struggled with test elements demanding greater flexibility and mobility (i.e. deep squat and active straight-leg raise), helping to account for the higher rate of dysfunction among males in this targeted age group (Bar-Or, 2004; Ghasempoor et al., 2018; Viru et al., 1999).

We found two moderate associations between FMS and FM in this Irish adolescent population, as displayed in Table 3: (a) locomotor and overall FMS™, and (b) overall FMS and overall FMS™, providing reasonable evidence for the relatedness of these two concepts*.* The correlation between composite FMS^TM^ and overall FMS locomotor skills (*r* = .30, *p* < .001) and associations between composite FMS locomotor skills and the deep squat FM (*r *= .18, *p* < .001) and shoulder mobility FM (*r* = .17, *p* < .001) may have been due to a high level of thoracic mobility underlying the biomechanics of both FMS locomotor skills and the FMS^TM^ tests of FM (Cook et al., 2014; Mason et al., 2016). Several FMS^TM^ assessments evaluate thoracic mobility - namely the deep squat, in-line lunge, and shoulder mobility exercises – while all FMS locomotor skills demand thoracic mobility (Ditcharles et al., 2017). In addition, ankle mobility, which is critical to performance of FMS^TM^ tests such as the deep squat and in-line lunge, is a key component of locomotor skills such as jumping (O’Brien et al., 2021; Papaiakovou, 2013). Collectively, these past and present findings suggest links between locomotor skills and composite FMS^TM^ performance, giving evidence of their relatedness that requires a deeper component analysis.

We found a small association (*r *= .224, *p* < .001) between the composite FMS^TM^ score for FM and FMS static balance, as measured by the Get Skilled Get Active tool (i.e. standing on one leg, with non-standing leg bent behind the body, and arms stretched to the side of the body). Kramer et al. (2019) reported moderate correlations between composite FMS^TM^ scores and dynamic balance among (males: *r* = 0.42; *p *< .01) and females (*r* = .41; *p* < .01), as measured by the Y-Balance test. The associations found across studies with both static and dynamic balance measures indicate that balance may serve as a key requirement for adequately performing tasks of the FMS^TM^ (Cook et al., 2006b; Kramer et al., 2019; Silva et al., 2019).

A moderate correlation between overall gross FMS and overall FM on the FMS™ composite score was reported (*r* = .375, *p* < .001). This association may arise from biomechanical similarities between certain FM and FMS (e.g., between squat flexion and the preparatory and landing phases of the vertical jump) which may account for the relatedness observed between FMS and FM in this study (Tompsett et al., 2014). While this study and others have shown small to moderate associations between locomotor and stability FMS constructs and FM through FMS^TM^ composite scores, further research and deeper analyses are necessary to determine if other underlying factors may contribute to the relatedness and associations observed between FMS and FM (O’Brien et al., 2021).

This study has highlighted movement deficiencies among Irish youth in FMS, low female proficiency in object-control skills, and low proficiency of males in locomotor skills. Additionally, we found dysfunctional movement scores in activities associated with flexibility and mobility among males, and dysfunctional movement scores in activities requiring upper-body core strength among females. While physical education curricula is not the sole contributor to these movement skill issues, our findings suggest that there are gaps in Irish physical education curricula that may not be optimally developing particular skills among students. To promote object-control skills among females, several studies have cited the importance of providing girls with autonomous, feasible challenges, and interesting learning experiences in both physical education and sport (Cowley et al., 2021; 2022; Farmer et al., 2017). For males, promoting locomotor-based play and such curriculum strands as dance and gymnastics should nurture their deficiencies in flexibility and mobility to help them develop a rounded skillset that meets their activity needs (Coulter et al., 2020; Garcia-Pinillos et al., 2018). Ultimately, however, a physical education environment that invites high participation, enjoyment and a wide array of games and exercise opportunities (as well as embracing diversity across all strands of the curricula such as health-related fitness, aquatics, and adventure education) is essential to the requisite development of both strength and flexibility skills among male and females (Dudley, 2015; Kriellaars et al., 2019). It is within the best interests of physical education stakeholders to provide a well-rounded curriculum for their students in their efforts to promote healthy and active individuals, with diverse movement abilities, in addition to strong social and teamwork skills (Belton et al., 2022; Cairney et al., 2019; Smith et al., 2021).

### Limitations and Directions for Further Research

A potential limitation of this research was that all participants were selected from Cork city in the urban and suburban area and socio-economic status was not controlled, meaning that these participants may not be representative of Irish adolescents from rural environments or all socioeconomic backgrounds. The sparse number of movements we used in assessing FMS stability is also a limitation, as this FMS category showed less performance variation than the object-control and locomotor FMS subsets. A more thorough examination of the link between the stability FMS subset and FM as measured by FMS™ may have been possible if the stability FMS subset had been more thoroughly examined through a combination of static and dynamic balance tests (e.g., those included in the Körperkoordinations test für Kinder, such as walking backwards on a balance beam, and moving sideways on wooden boards). This was not possible owing to our time constraints during data collection. While this study serves as a preliminary investigation into the relatedness between FMS and FMS^TM^, future investigators might cross validate and extend these results with a more comprehensive examination (possibly through divergent and convergent validity assessments or through confirmatory factor analysis). Additionally, longitudinal studies would permit an analysis of cause and effect relationships between learning experiences and FMS and FM. Comparative FMS^TM^ data from sporting and non-sporting adolescents would also assist data interpretation.

## Conclusion

In this study, a large number of Irish adolescents displayed poor FMS and FM proficiency compared to previously established values. Moderate associations between FMS and FM measures in these cross-sectional data further suggests a need for further research into these MC components. Both FMS and FM represent movements that are important for PA throughout the lifespan, however, they must continue to be viewed separately and assessed using only validated testing measurements. Given the low levels of adolescent FMS and FM proficiency we observed in these Irish adolescents, societal interventions targeting the development of FMS and FM proficiency in early adolescent Irish youths are apt to be beneficial for improving later PA and health. Sex-specific interventions may be needed to ameliorate specific deficiencies observed in males and females, respectively.
